# AlphaPart—R implementation of the method for partitioning genetic trends

**DOI:** 10.1186/s12711-021-00600-x

**Published:** 2021-03-18

**Authors:** Jana Obšteter, Justin Holl, John M. Hickey, Gregor Gorjanc

**Affiliations:** 1 Department of Animal Science, Agricultural Institute of Slovenia, Hacquetova ulica 17, 1000 Ljubljana, Slovenia; 2 The Pig Improvement Company, Genus plc, 100 Bluegrass Commons Blvd., Ste 2200, Hendersonville, TN 37075 USA; 3 The Roslin Institute and Royal (Dick) School of Veterinary Studies, University of Edinburgh, Easter Bush, Midlothian, EH259RG UK

## Abstract

**Background:**

In this paper, we present the AlphaPart R package, an open-source implementation of a method for partitioning breeding values and genetic trends to identify the contribution of selection pathways to genetic gain. Breeding programmes improve populations for a set of traits, which can be measured with a genetic trend calculated from estimated breeding values averaged by year of birth. While sources of the overall genetic gain are generally known, their realised contributions are hard to quantify in complex breeding programmes. The aim of this paper is to present the AlphaPart R package and demonstrate it with a simulated stylized multi-tier breeding programme mimicking a pig or poultry breeding programme.

**Results:**

The package includes the main partitioning function AlphaPart, that partitions the breeding values and genetic trends by pre-defined selection paths, and a set of functions for handling data and results. The package is freely available from the CRAN repository at http://CRAN.R-project.org/package=AlphaPart. We demonstrate the use of the package by partitioning the nucleus and multiplier genetic gain of the stylized breeding programme by tier-gender paths. For traits measured and selected in the multiplier, the multiplier selection generated additional genetic gain. By using AlphaPart, we show that the additional genetic gain depends on accuracy and intensity of selection in the multiplier and the extent of gene flow from the nucleus. We have proven that AlphaPart is a valuable tool for understanding the sources of genetic gain in the nucleus and especially the multiplier, and the relationship between the sources and parameters that affect them.

**Conclusions:**

AlphaPart implements the method for partitioning breeding values and genetic trends and provides a useful tool for quantifying the sources of genetic gain in breeding programmes. The use of AlphaPart will help breeders to improve genetic gain through a better understanding of the key selection points that are driving gains in each trait.

## Background

Breeding programmes improve populations for a set of traits by selecting and mating genetically superior individuals. Population improvement can be measured with a genetic trend calculated by averaging estimated breeding values by year of birth [1, 2]. While sources of the overall genetic gain are generally known, their realised contributions are hard to quantify in complex breeding programmes due to many interacting processes. García-Cortés et al. [3] proposed a method for such analyses. In summary, the method uses pedigree information to first partition the breeding values into a parent average and a Mendelian sampling term: ${a}_{i}=1/2{a}_{s}+1/2{a}_{d}+{w}_{i}$ [4], where ${a}_{i}$, ${a}_{s}$, and ${a}_{d}$ are the breeding values of the individual, its sire, and its dam, respectively, and ${w}_{i}$ is the individual’s Mendelian sampling term. The parent average represents the expected breeding value of progeny given the breeding values of parents and the Mendelian sampling term represents the deviation arising from recombination and segregation of parental chromosomes. The partitioning method then allocates Mendelian sampling terms to the selection path that generated it. For example, assuming a small trio pedigree with two parents and a female progeny, and specifying gender with two levels (males and females) as the selection path variable, we can write ${a}_{i}=1/2{a}_{s}+1/2{a}_{d}+{w}_{i}=\left(1/2{a}_{d}+{w}_{i}\right)+1/2{a}_{s}={a}_{if}+{a}_{im}$, where ${a}_{if}$ denotes the contribution of the female path and ${a}_{im}$ denotes the contribution of the male path. Since individual $i$ is a female, we assigned her Mendelian sampling term ${w}_{i}$ to the contribution of females as we did with half of the dam’s breeding value ($1/2{a}_{d}$). We assigned half of the sire’s breeding value to the contribution of males. Alternatively, assuming that the sire is imported, then an interesting path specification can be made that separates contributions from domestic versus imported sources, which can be partitioned similarly to the gender example. In general, we can write a vector of breeding values as a linear combination of Mendelian sampling terms of individuals and their ancestors: $\mathbf{a}=\mathbf{T}\mathbf{w}$, where $\mathbf{T}$ is a triangular matrix of expected gene flow between ancestors and individuals [4, 5] and $\mathbf{w}$ is a vector of Mendelian sampling terms. The method of García-Cortés et al. [3] uses a path variable to partition the gene flow matrix $\mathbf{T}={\mathbf{T}}_{1}+{\mathbf{T}}_{2}+\dots +{\mathbf{T}}_{\mathrm{p}}$ and with this partitions breeding values by selection paths ${\mathbf{a}=(\mathbf{T}}_{1}+{\mathbf{T}}_{2}+\dots +{\mathbf{T}}_{\mathrm{p}})\mathbf{w}={\mathbf{a}}_{1}+{\mathbf{a}}_{2}+\dots +{\mathbf{a}}_{\mathbf{p}}$**)**, where ${\mathrm{T}}_{\mathrm{i}}$ describes the gene flow for a selection path $\mathrm{i}$. Aggregating these partitions by other variables (such as year of birth, countries, gender, etc.) is a powerful way to analyse sources of genetic gain.

The partitioning method has been used in a number of cases. Gorjanc et al. [6, 7] estimated contributions of breeding programmes in different countries to country-specific and global genetic trends in the Brown-Swiss and Holstein populations. Špehar et al. [8] estimated contributions of domestic and foreign selection paths to genetic gain of Croatian Simmental cattle, and Škorput et al. [9] estimated such contributions to genetic gain in two pig breeds in Croatia. The latter study also extended the analysis by accounting for the uncertainty of estimated breeding values and partitions [2]. However, these studies used bespoke implementations of the partitioning method, for which no open-source software exists.

The aim of this paper is to present the AlphaPart R package that implements a method for partitioning breeding values and genetic trends. We demonstrate the package with an example in which we partition the genetic gain of a small population for which genetic material is imported. Next, we demonstrate the package by partitioning the genetic trends of a stylized multi-tier breeding example usually applied in pig and poultry breeding.

## Implementation

AlphaPart is an R package available from the CRAN repository at https://CRAN.R-project.org/package=AlphaPart and thus easily installed via install.packages(“AlphaPart”) and loaded with library(AlphaPart) command inside R. We developed the package with the aim to create a user-friendly implementation of the method for partitioning breeding values and genetic trends. The only input required from the user is an initial data frame. All the subsequent functions in the analysis pipeline accept the output of a preceding function.

First, we demonstrate the standard AlphaPart analysis pipeline in an example session by analysing genetic gain in a breeding programme that imports genetic material from another population. Next, we demonstrate the usefulness of AlphaPart by analysing the dynamics of genetic gain in a multi-tier breeding programme. The simulation code for the generation and analysis of both datasets is available in the GitLab repository https://git.ecdf.ed.ac.uk/HighlanderLab_public/jobsteter_alphapart.

### Example session of analysing a breeding programme using import

Here, we demonstrate the functionality of AlphaPart on a simulated example. Consider a case of a small population (Population 1) that imports genetic material from two larger populations (Populations 2 and 3) that have higher genetic gains per unit of time. Populations 2 and 3 achieve higher genetic gains with higher accuracy of selection (h2 = 0.9 vs. h2 = 0.7 in Population 1). In addition, Population 3 benefits from a higher intensity of male selection (2.89 vs. 2.67 in Populations 1 and 2). In this simulation, we assume the presence of genotype-by-environment interactions and genetic correlations of a trait in Population 1 with a trait in Population 2 and a trait in Population 3 of 0.9 and 0.8, respectively, and we assume 20 generations of selection of males on phenotypic values. In generations 1 to 10, we perform selection within these populations without import and in generations 11 to 20, Population 1 imports 10% of semen from Population 2 and 10% of semen from Population 3. To optimize the breeding strategy in Population 1, we would like to quantify how much genetic gain between generations 11 and 20 stems from each of the populations. Hence, we partition the breeding values by variable “population of origin”. To analyse this situation, we used stochastic simulation to generate the dataset. Details of the simulation are provided in Additional file 1: Figure S1.

### Preparing the data

**Fig. 1:**
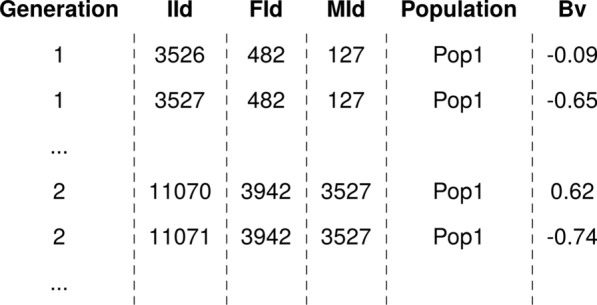
Example input data frame for partitioning analysis. The data frame shows the first few rows of individual’s generation, identification (IId), father’s identification (FId), mother’s identification (MId), population and breeding value (Bv)

The main input for the analysis is a data frame that holds pedigree information with individual/sire/dam or individual/sire/maternal-grandsire identification, a time-ordering variable such as year of birth, partition variable (path), and breeding values for one or multiple traits. The package provides functions to pre-process the input data, such as for correcting missing or incorrect years of birth and setting the base population of the pedigree. For this example session we prepared the input data frame PedEval containing the year (generation) of birth (Generation), identifications for individuals and their parents (IId, FId, and Mid), population or origin (Population), and breeding values (Bv) for the trait expressed in Population 1 (Fig. 1).

The missing or erroneous years of birth in the input data affect the accuracy of partitioning. The package allows us to impute missing or correct erroneous years of birth with the pedFixBirthYear() function. Since we are using simulated data in this example, we have no missing or erroneous generations of birth. If we had, we would run:
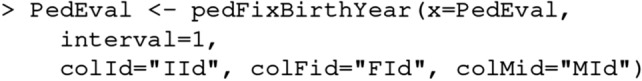


The pedFixBirthYear() function requires the input data frame as described above (Fig. 1) that holds at least individual, father and mother identification, and the year of birth. It also requires the generation interval via the interval argument. The function computes the missing birth years either (i) based on offspring by adding the generation interval to the birth year of the oldest offspring to obtain the parent birth year (argument down set to FALSE); or (ii) based on parents by subtracting the generation interval from the birth year of the youngest parent to obtain offspring birth year (argument down set to TRUE). The output of the function is a data frame with corrected years of birth.

Our data frame contains information on 20 generations of selection, for 10 of these, selection is performed within each of the populations, and for the 10 following generations, genetic material from Populations 2 and 3 is imported into Population 1. If we want to consider only the generations in which we perform import, that is generations 11 to 20, we use the function pedSetBase() to rebase the pedigree:

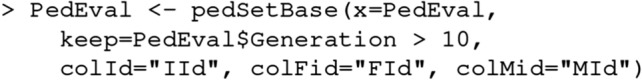



The pedSetBase() function requires the input data frame as described above (Fig. 1) that holds at least individual, father and mother identification, and the year of birth. It also requires instructions on where to rebase the pedigree via the keep argument. The function removes all the individuals that do not meet the condition, including their role as parents. The output of the function is a data frame with an adjusted set of individuals.

### Partitioning analysis

We partition the breeding values with the main function of the package, AlphaPart(). To partition breeding values (Bv) into contributions of selection in Population 1 (domestic source), Population 2 (imported source) and Population 3 (imported source) we use:

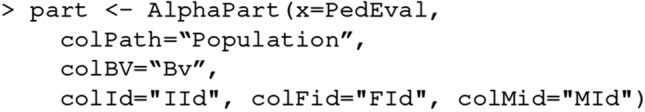



The function AlphaPart() requires the input data frame as described above (Fig. 1). Following the method described by García-Cortés et al. [3], the function recurses the pedigree from the oldest to the youngest individual and calculates for each individual its parent average and Mendelian sampling terms for the trait. Then, it assigns half of the parent average term to each of the parents’ paths and Mendelian sampling term to the individual’s path. For the founders, the function assigns their entire breeding values to the founders’ paths. The function can also conveniently partition breeding values for multiple traits by specifying a vector of variables, say colBV = c(“Bv1”, “Bv2”). This specification triggers simultaneous partitioning of multiple vectors (breeding values) in the input data frame, but each vector is partitioned independently. The multiple trait option can also serve to partition samples from the posterior distribution of breeding values to quantify uncertainty due to estimation from the available data [2, 9]. To speed-up calculations, we use C++ and simultaneous partitioning of multiple traits (where needed). The function can also simultaneously partition and summarize path contributions by a grouping variable (for example generation) provided via the colBy argument, which is a useful computational speed-up for huge pedigrees. Alternatively, we subsequently use the summary.AlphaPart() function to summarize the partitions as shown below.

**Fig. 2:**
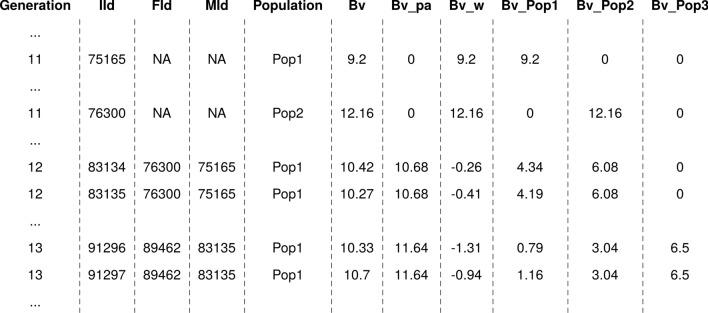
Example output data frame from partitioning analysis with the AlphaPart() function. The data frame holds for each individual all the columns from the input data frame (Fig. 1) as well as parent average (Bv_pa), Mendelian sampling term (Bv_w), and breeding value partitions (Bv_Pop1, Bv_Pop2 and Bv_Pop3)

The output of the AlphaPart() function is an object of either a data frame with partitioned breeding values (AlphaPart class) or partitioned and summarized breeding values (summaryAlphaPart class). In either case, the output is a list with an info element and an element with partitioned breeding values for each of the traits. The info element is a list with information on the path variable (path), number of paths (nP), path labels (lP), number of traits (nT), trait labels (lT), and putative warnings (warn). The trait element is a data frame, named after the analysed trait (Fig. 2), and holds, for each individual, all the columns from the input data frame as well as parent average (Bv_pa), Mendelian sampling term (Bv_w), and breeding value partitions (Bv_Pop1, Bv_Pop2 and Bv_Pop3).

In Fig. 2, we see that individuals 75165 (Population 1, breeding value 9.20) and 76300 (Population 2, breeding value 12.16) are treated as founders without pedigree information. Thus, their entire breeding value is assigned to their Mendelian sampling term and to their populations of origin. The breeding value of their offspring, individuals 83134 and 83135, are partitioned into parent average and Mendelian sampling terms and they are also partitioned by the population of the animal’s origin. For example, breeding value of 83134 is 10.42 with parent average 10.68 = (9.20 + 12.16)/2 and Mendelian sampling term − 0.26. Since parents are from Populations 1 and 2 and their progeny is from Population 1, we can partition the offspring breeding value, 10.42, also into the contribution of Population 1, 9.20/2—0.26 = 4.34, and contribution of Population 2, 12.16/2 = 6.08. Offspring 83135 is mated with individual 89462 from Population 3 with a breeding value of 13.01 (not shown). The breeding values of their offspring, individuals 91296 and 91297, are therefore partitioned into contributions from three populations.

### Analysing the results

To summarise the individual partitions of breeding values, the package includes functions to summarize and/or combine paths by a grouping variable, subset the partitioning results and visualise these summaries.

An interesting measure is the trend of mean breeding values (genetic trend) and path partitions (partial genetic trends) through time. To summarize the breeding values and the partitions by generation, we use the summary.AlphaPart(). Since we are interested in the genetic gain of Population 1, we use the subset option to filter out Population 1 individuals:

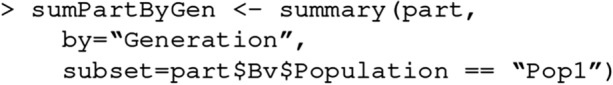



The input for the summary.AlphaPart() is the output of the partitioning analysis (AlphaPart class). The function also requires a variable that allows us to summarize the breeding values and their partitions via the by argument. In the case above, we are summarizing by generation, but depending on the analysis this could be any variable (for example, gender, population, … and their combinations). By default, the function summarizes the mean of the breeding values of the analysed trait, but the user can specify any R function via the FUN argument. The function can also summarize only a subset of the object by specifying which individuals to keep. The user passes the condition as a vector of logical values via the subset argument as shown above.

**Fig. 3:**
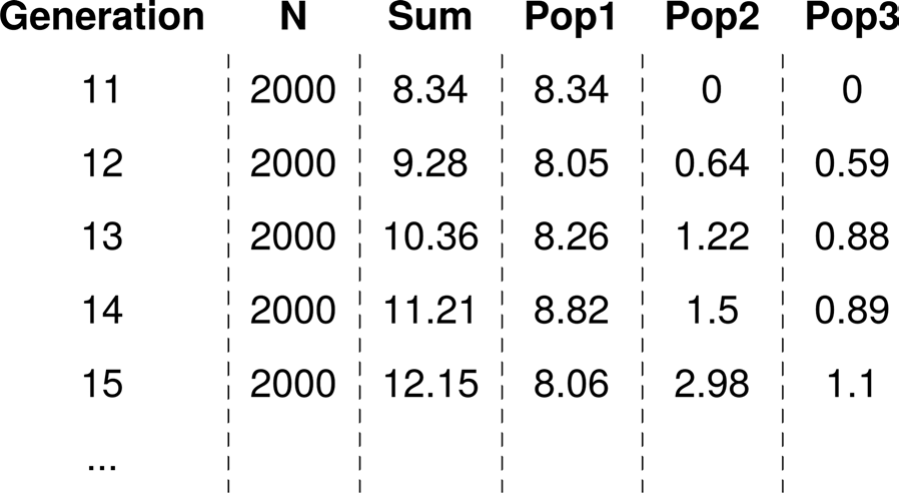
Example output data frame of summarized partitions obtained with the summary.AlphaPart() function. The data frame holds information on generation, number of data points (N), the total genetic gain (Sum) and mean contributions of the three populations (Pop1, Pop2, and Pop3)

The output of the summary.AlphaPart() function is a data frame that holds summarized breeding values and their partitions for the trait (summaryAlphaPart class). It is a list with an info element and one element for each partitioned trait. The summary element, named after the analysed trait (Fig. 3), contains the grouping variable (Generation), number of individuals per level (N), and output of the summary function applied to the breeding values (Sum) and its partitions (Pop1, Pop2, and Pop3).

We can use package functions to further manipulate the partitioned or summarized results. If we are only interested in the contributions of Populations 1 and 2, we can filter out the summary results with the AlphaPartSubset() function:

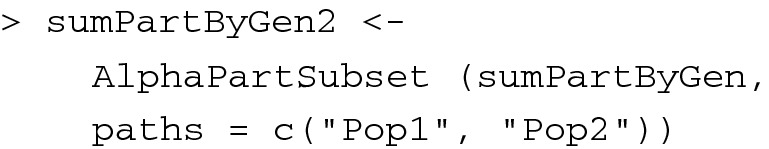



The AlphaPartSubset() function takes the output of either the partitioning (AlphaPart class) or summarizing (summaryAlphaPart class) analysis, and a character vector of paths to keep via the paths argument. The output of the function is a subset of the dataset of the input class.

We could also be interested in only the contribution of domestic vs. imported sources and not of specific populations. To this end, we can combine the contributions of imported sources, i.e. from Populations 2 and 3, with the AlphaPartSum() function and compare the contributions of Population 1 (named Domestic) vs. the combined contributions of imported sources (named Import):

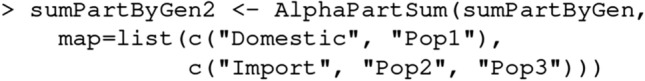



The input for AlphaPartSum() can be the output of either the partitioning (class AlphaPart) or summarizing (class summaryAlphaPart) analysis. The function also takes a list of paths to sum via the map argument. Each element of this list contains the name of the newly created combined path followed by the names of the paths to combine.

Lastly, we can plot the summarized partitions with the plot.summaryAlphaPart() function:

> plot(sumPartByGen)

**Fig. 4:**
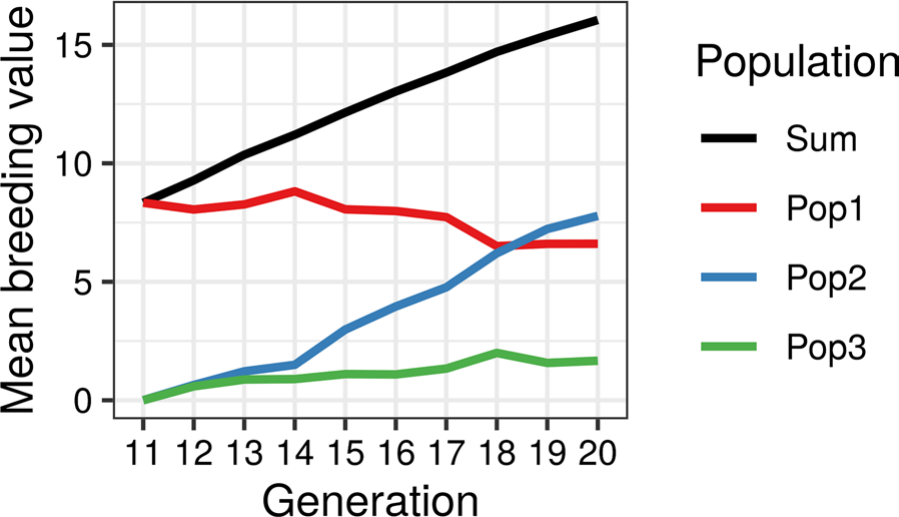
Example output plot of summarized partitions obtained with the plot.summaryAlphaPart() function. The plot shows the overall genetic trend and its partition into the contributions of domestic selection (Pop1) and import from two populations (Pop2 and Pop3) by generation

The input for the plotting function must be a summarized dataset (class summaryAlphaPart), which could have been further subsetted or in which some of the paths have been combined. The output is a list (plotSummaryAlphaPart class) containing one plot for each partitioned trait. We show the output plot in Fig. 4.

### Simulated multi-tier breeding example

We applied the AlphaPart R package on a simulated example of a multi-tier pig breeding programme. Our aim was to examine the gene flow between nucleus and multiplier and the contribution of nucleus and multiplier selection on genetic gain in each tier. Breeders select in the nucleus and multiply this improvement in the multiplier to supply a large number of breeding animals for commercial purposes. The multiplier generally has a lower genetic mean than the nucleus due to a time-lag in the gene flow. However, animals with very high breeding values for some traits can be observed in the multiplier tier. We simulated a multi-tier breeding programme and used AlphaPart to partition the genetic trend of true breeding values by a tier-gender variable to quantify sources of genetic gain in the nucleus and the multiplier.

**Fig. 5:**
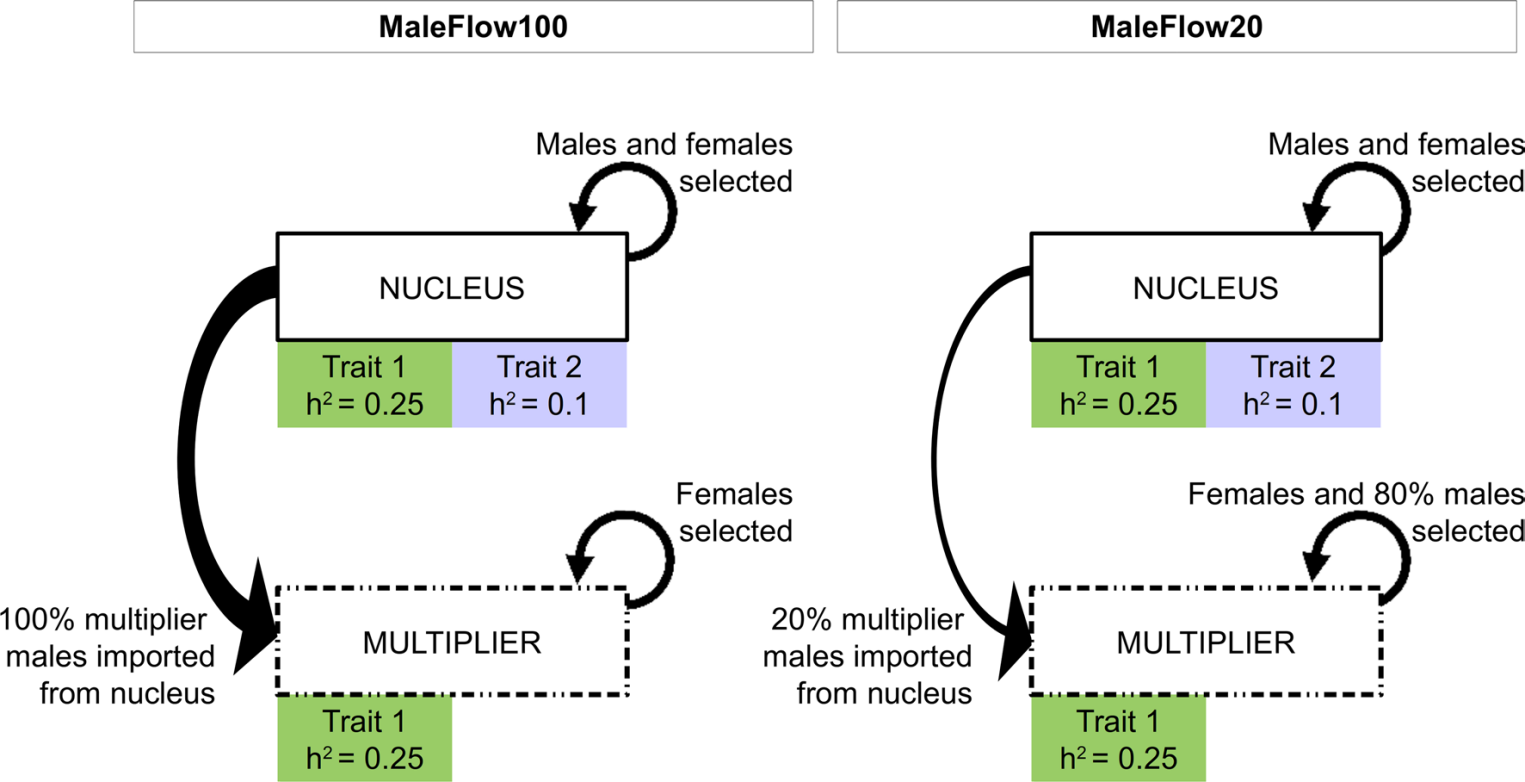
Design of the simulated stylized multi-tier breeding programmes. We simulated two scenarios with a closed nucleus and a directional flow of animals from the nucleus into the multiplier. The scenarios differ in the percentage of multiplier males imported from the nucleus

We used the AlphaSimR package [10] for stochastic simulation of a multi-tier breeding programme for a single breed with a closed nucleus and a flow of animals from the nucleus into the multiplier (Fig. 5). We simulated 40 generations of selection on two traits. Trait 1 had a heritability of 0.25 and trait 2 had a heritability of 0.10. The traits were influenced by the same set of causal loci, but the effect of these loci on the two traits were uncorrelated. We measured both traits in the nucleus and only trait 1 in the multiplier. We selected on an index with equal weights on the estimated breeding values for the two traits. We split the simulation into an initial burn-in period of 20 years to achieve a population equilibrium and a subsequent 20-year period of genetic evaluation and selection.

In the burn-in period, we simulated only the nucleus and we selected animals based on the index of phenotype values for both traits. We selected 25 males and 500 females in each generation and randomly mated them to produce a new generation of 6000 progeny (12 per mating). At the end of the burn-in, we generated 5000 females to seed the multiplier.

In the evaluation, we simulated both the nucleus and the multiplier and selected animals within each tier based on the index of estimated breeding values for both traits (Fig. 5). In the nucleus, we selected 25 nucleus males and 500 nucleus females in each generation and randomly mated them to produce a new generation of 6000 progeny (12 per mating). In the multiplier, we selected 750 multiplier females in each generation and randomly mated them to a set of males to produce a new generation of 9000 progeny (12 per mating). To quantify the effect of selection in the multiplier on genetic gain, we defined the set of males as either (1) the 25 best nucleus males (MaleFlow100 scenario—100% of males are from the nucleus) or (2) the 25 best nucleus males and 100 best multiplier males (MaleFlow20 scenario—20% of males are from the nucleus and the number of females per male is smaller). We emphasise that nucleus males were used in the nucleus and multiplier without delay to reduce genetic lag between them.

We estimated the breeding values for each trait independently before each nucleus or multiplier selection decision. We ran a pedigree-based model implemented in blupf90 [11] and used all available data from evaluation generations. The model included the mean as a fixed effect and animal breeding values as a random effect modelled hierarchically with pedigree.

Finally, we partitioned the true breeding values and genetic trends with AlphaPart as demonstrated above. We used the AlphaPart() function to partition standardized true breeding values from the 20 evaluation generations by the tier-gender variable and the summary.AlphaPart() function to summarize the partitions by generations to quantify the contribution of each tier-gender level to genetic trend in the nucleus and the multiplier.

We repeated the simulation 10 times and measured the genetic trend separately in the nucleus and multiplier. We present standardized true breeding values and genetic trends, as well as their partitions with the mean set to zero and the genetic standard deviation set to one in generation 20. We chose to present true (instead of estimated) breeding values to assess the true sources of genetic gain.

## Results

The results show partitions of true breeding values and genetic trends in the nucleus and multiplier obtained with AlphaPart for the two simulated stylized multi-tier breeding scenarios. Partitioning showed that we can explain the situation with very high breeding values in the multiplier by the extent of nucleus-multiplier gene flow as well as accuracy and intensity of multiplier selection. For each scenario, we first describe the distribution of true breeding values in the nucleus and multiplier in generation 40 of one replicate. Next, we explain the sources of the observations by partitioning the nucleus and multiplier genetic trend and averaging the results across ten replicates. The distributions of partitioned true breeding values for one replicate are shown in Additional file 2: Figure S2 and Additional file 3: Figure S3.

### MaleFlow100 scenario

#### Distribution of breeding values

**Fig. 6:**
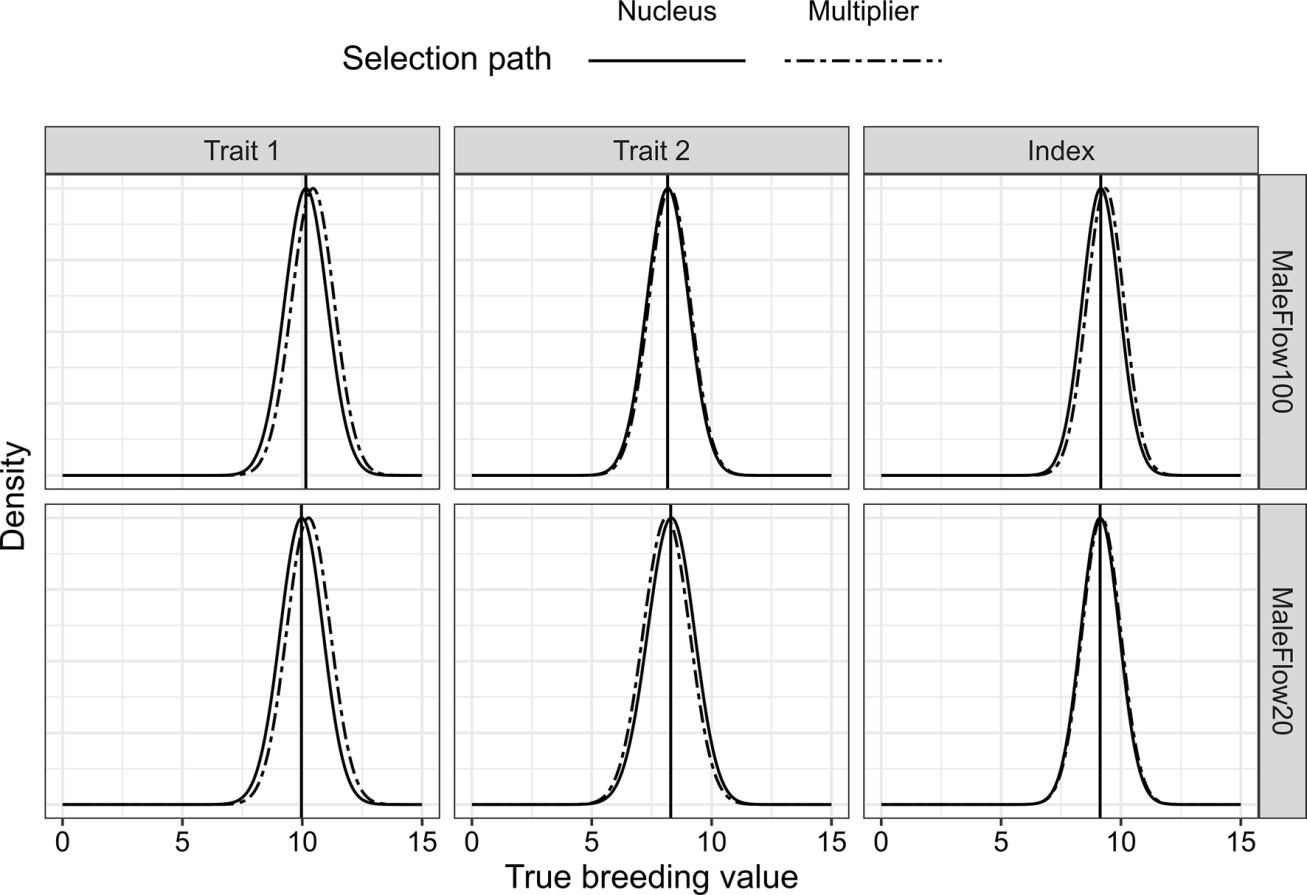
Distribution of true breeding values by trait, tier, and scenario. We show scaled densities in generation 40 of one simulation replicate. MaleFlow100 uses only nucleus males in the multiplier, and MaleFlow20 uses nucleus and multiplier males in the multiplier. Trait 1 is measured in the nucleus and the multiplier, while trait 2 is measured only in the nucleus. Black vertical lines represent the nucleus mean breeding value for a trait

In the MaleFlow100 scenario, the multiplier had a higher genetic merit on average than the nucleus for trait 1 and trait 2 as shown in Fig. 6 with the distribution of true breeding values in the nucleus and the multiplier by trait in generation 40 of one replicate. The multiplier had a higher genetic merit on average and hence produced animals with a higher breeding value than the nucleus for both traits, which was reflected in a higher index value as well.

#### Partitioning the true breeding values and genetic trend

**Fig. 7:**
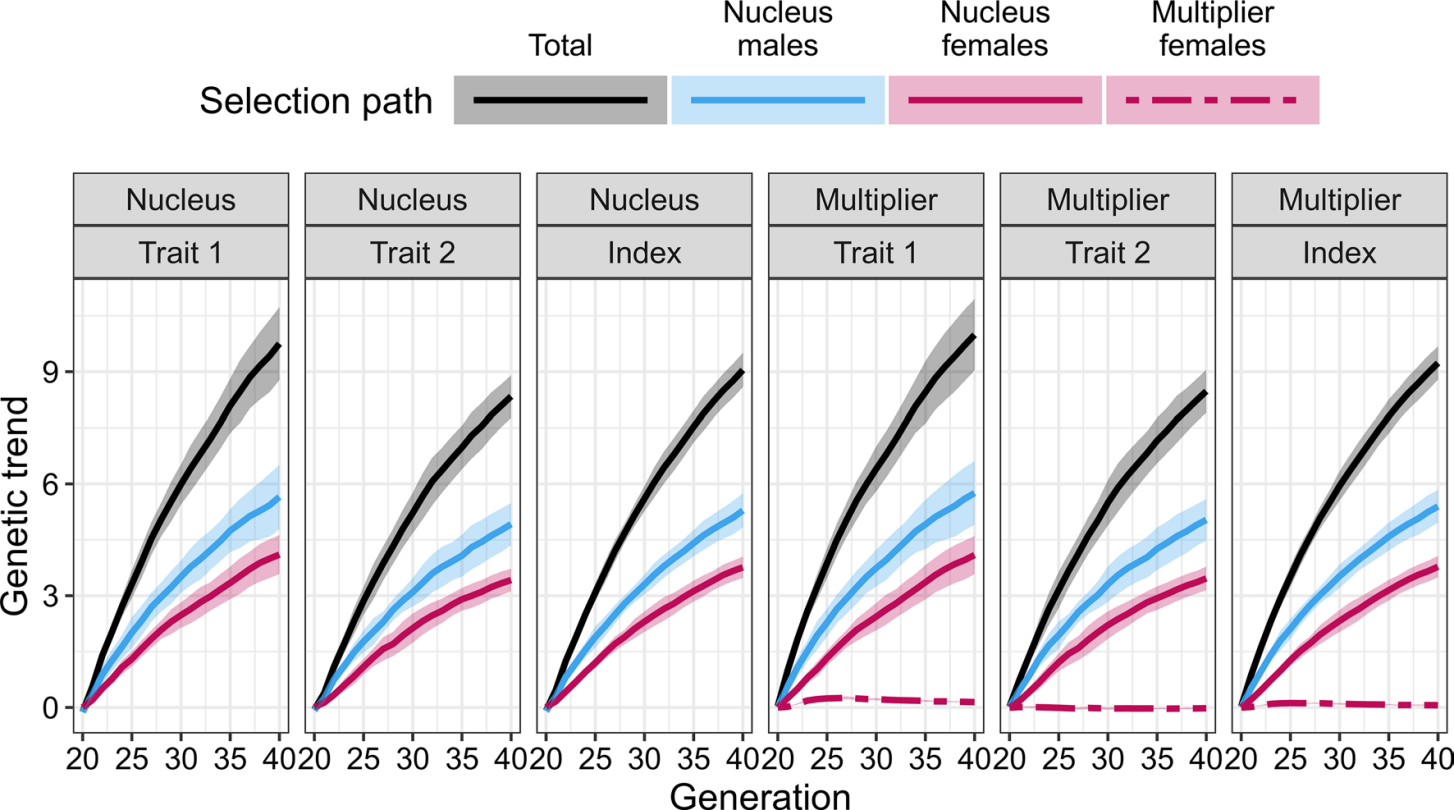
Partitioning of genetic trend by tier-gender in the MaleFlow100 scenario. The scenario uses nucleus males in the multiplier. Trait 1 is measured in the nucleus and the multiplier, while trait 2 is measured only in the nucleus

The partitioning showed that the higher genetic gain in the multiplier compared to the nucleus for trait 1 was due to an additional contribution from selection of multiplier females as shown in Fig. 7 with the genetic trends in the nucleus and multiplier by trait and their partitions summarised over 10 replicates. As expected, the nucleus genetic gain stemmed completely from the selection of nucleus males and nucleus females. The selection of nucleus males contributed more to the genetic gain than the selection of nucleus females. The mean genetic gains at generation 40 in the nucleus were 9.75 and 8.34 for traits 1 and 2, respectively, with male selection contributing 5.65 for trait 1 and 4.92 for trait 2, and female selection contributing 4.10 for trait 1 and 3.42 for trait 2.

In the multiplier, the average genetic gain for trait 1 was higher than in the nucleus. This increase was driven by two sources. First, nucleus males made a larger contribution to the multiplier than to nucleus genetic gain, since they contributed directly by fathering the multiplier animals and indirectly through subsequent selection of their genes in future generations. Second, multiplier female selection made a non-zero contribution. The mean genetic gain at generation 40 in the multiplier for trait 1 was 10.00 with nucleus males contributing 5.75, nucleus females 4.09, and multiplier females 0.14. The mean genetic gain and its path partitioning at generation 40 for trait 2 were similar in the multiplier and the nucleus. The distributions of partitioned true breeding values for one replicate are shown in Additional file 2: Figure S2.

### MaleFlow20 scenario

#### Distribution of breeding values

In the MaleFlow20 scenario, the genetic merit was higher in the multiplier than the nucleus for trait 1, but lower for trait 2 as shown in Fig. 6 with the distribution of true breeding values in nucleus and multiplier by trait in generation 40 of one replicate. Again, we observed animals with higher breeding values for trait 1 in the multiplier than in the nucleus, with an even larger difference than in the MaleFlow100 scenario. We did not observe the same phenomena for trait 2.

#### Partitioning the true breeding values and genetic trend

**Fig. 8:**
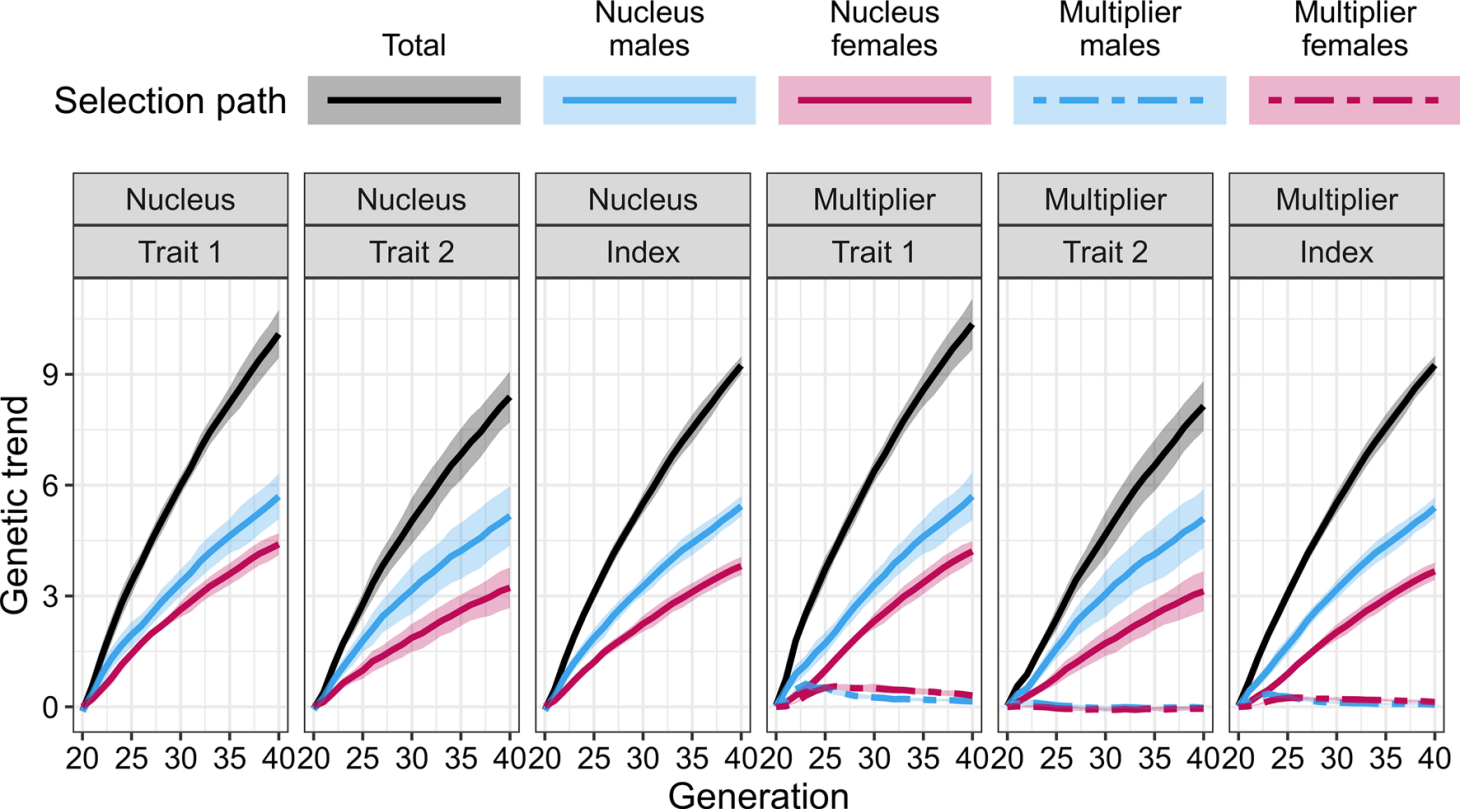
Partitioning of the genetic trend by tier-gender in the MaleFlow20 scenario. The scenario uses nucleus and multiplier males in the multiplier. Trait 1 is measured in the nucleus and the multiplier, while trait 2 is measured only in the nucleus

The partitioning revealed, that selection of multiplier males and females further increased the genetic gain for trait 1 in the multiplier compared to the nucleus, but decreased the genetic gain for trait 2 as shown in Fig. 8 with the genetic trends in the nucleus and multiplier by trait and their partitions summarised over 10 replicates. As in MaleFlow100 scenario, the nucleus genetic gain stemmed from selection of nucleus males and females. The mean genetic gains at generation 40 were 10.09 and 8.39 for traits 1 and 2, respectively, with nucleus males contributing 5.69 for trait 1 and 5.17 for trait 2, and nucleus females contributing 4.40 for trait 1 and 3.22 for trait 2.

In the multiplier, the genetic gain was again higher than in the nucleus, but only for trait 1. This higher genetic gain was a result of non-zero contribution of multiplier female and male selection and a reduced contribution of nucleus females. In MaleFlow20, we reduced the use of nucleus males in the multiplier, which reduced the contribution of nucleus females via reduced nucleus-multiplier gene flow. For trait 2, the genetic gain in the multiplier was lower than in the nucleus due to a small average negative contribution of multiplier females and multiplier males and reduced contribution of nucleus females and nucleus males via reduced gene flow. The mean genetic gain at generation 40 in the multiplier was 10.36 for trait 1 and 8.14 for trait 2, with nucleus males contributing 5.70 for trait 1 and 5.09 for trait 2, nucleus females contributing 4.21 for trait 1 and 3.13 for trait 2, multiplier males contributing 0.15 for trait 1 and -0.03 for trait 2, and multiplier females contributing 0.30 for trait 1 and -0.05 for trait 2. The distributions of partitioned true breeding values for one replicate are shown in Additional file 3: Figure S3.

## Discussion

In this paper, we present AlphaPart, a freely available R package that implements the method for partitioning breeding values and genetic trends. We demonstrate the package on a simulated stylized multi-tier breeding example with a higher genetic trend for some traits in the multiplier compared to the nucleus. Following this, we organized the discussion into two parts: (i) advantages and disadvantages of the AlphaPart R package and (ii) partitioning results of the breeding example.

### AlphaPart

AlphaPart is a free implementation of the method for partitioning breeding values and genetic trends. The method and the package are valuable for deciphering and quantifying the sources of genetic gain in breeding programmes. The package is easy to use, since it streamlines the partitioning analysis into a few lines of R code. AlphaPart presents a holistic tool to perform a partitioning analysis, from preparing the input data—such as manipulating the pedigree data—to handling of results and plotting. The partitioning step is fast, even for large pedigrees, since the main partitioning function is recursive and implemented in C++.

AlphaPart is aimed at researchers who are interested in quantifying the sources of genetic gain in their breeding programmes in order to understand the dynamics of genetic gain, improve selection efficiency in certain partitions, assess the performance of different breeding actions, or to optimize investment. The accuracy of partitioning depends on the accuracy of the estimated breeding values and their Mendelian sampling terms, which are driven by the genetic parameters of the trait, the information available in the breeding programme structure used, and choice of the prediction model.

Future development of AlphaPart will include an extension of the partitioning method in three areas. The first extension will use genomic information to inform which genome regions and which specific haplotypes or alleles drive genetic change. The second extension will use the partitioning method to analyse contributions to changes in genetic variance in addition to the genetic mean. The third extension will simplify handling of uncertainty of path contributions when working with samples from posterior distributions [2, 9].

### Multi-tier breeding example

The multi-tier breeding example illustrated the investigative power of the partitioning method and the free AlphaPart implementation. Here, we discuss the sources of genetic gain in the two tiers of a breeding programme.

By partitioning the genetic trend in a simulated multi-tier breeding programme, we disentangled the observation that some multiplier animals have higher breeding values for some traits compared to the nucleus animals. While larger numbers of individuals and therefore recombinations in the multiplier can potentially reveal more variation and occasional outlying animals (due to an outstanding recombination), we expect lower breeding values in the multiplier due to time-lag between the nucleus and multiplier. In this study, we analysed a situation with no or limited genetic lag on the male side. We showed with partitioning that the gene flow from the nucleus into the multiplier was expectedly the main source of genetic gain in the multiplier.

However, the results also showed that selection in the multiplier can contribute genetic gain in addition to the gene flow from the nucleus. The multiplier outperformed the nucleus for trait 1, because with the 10,500 recorded multiplier animals there was a substantial amount of information for accurate multiplier selection that generated additional genetic gain. We emphasise that this result is also due to a limited time-lag between the nucleus and multiplier as we used the nucleus males in the nucleus and multiplier concurrently assuming artificial insemination. The partitioning of genetic trend for trait 1 showed that when we used only the nucleus males in the multiplier (MaleFlow100), the multiplier generated additional gain from two sources. First, compared to the nucleus, the contribution of the nucleus males increased because they contributed through the immediate gene flow and through the long-term gene flow contributions that influenced the selection of multiplier females. Second, the selection of multiplier females contributed as well. When we used both the nucleus males and the multiplier males in the multiplier (MaleFlow20), the multiplier generated further gain through a combination of the sources—the contribution of selection in multiplier females and males, and the decreased contribution of nucleus selection due to the reduced gene flow. This decrease was due to a smaller number of progeny per nucleus male in the multiplier compared to the MaleFlow100 scenario. In both scenarios, we observed a trend of decreasing contribution of multiplier selection over generations, although the average multiplier contribution was always above zero. Since we partitioned breeding values with generation 20 as a base generation, the parent average and Mendelian sampling terms for multiplier animals in generation 20 were assigned to the multiplier path. Over the generations, the nucleus and multiplier contributions converged since the pedigree used in next generations accounted for the origin of the nucleus males. This shows the importance of proper base population specification (including unknown parent groups) for meaningful partitioning. This long-term dynamic of contributions is related to the dynamics of “long-term genetic contributions” in the context of genetic gain and inbreeding [12, 13], but it should be noted that the “long-term genetic contributions” are trait agnostic (depend only on the pedigree). On a related note, with the implemented method in AlphaPart, we can evaluate (long-term) genetic contributions by setting breeding values to 1 for all animals and partitioning the breeding values by paths [6].

On the contrary, trait 2 was not measured in the multiplier and had a comparable or smaller genetic trend in the multiplier than in the nucleus. For trait 2, the multiplier animals were selected only on estimated parent average, which resulted in low accuracy selection. In the MaleFlow100 scenario, this low accuracy selection resulted in a null contribution of multiplier females to the genetic trend for trait 2 and comparable genetic trends between the nucleus and the multiplier. In the MaleFlow20 scenario with a reduced nucleus-multiplier gene flow, this low accuracy selection resulted in the reduced genetic gain for trait 2.

## Conclusions

AlphaPart R package is a freely available software for partitioning breeding values and genetic trends. Use of AlphaPart will help breeders to better understand sources of genetic gain and improve their breeding programmes.

## Supplementary Information

**Additional file 1: Figure S1.** Example session of analysing a breeding programme using import—simulation details.

**Additional file 2: Figure S2. **Distribution of true breeding values and their partitions by trait, year, and tier in the MaleFlow100 scenario. We show scaled densities of partitions in years 23 and 40 of one simulation replicate. MalerFlow100 uses only nucleus males in the multiplier. Trait 1 is measured in the nucleus and the multiplier, while trait 2 is measured only in the nucleus. Black vertical lines represent the nucleus mean breeding value for a trait in a year.

**Additional file 3: Figure S3. **Distribution of true breeding values and their partitions by trait, year, and tier in the MaleFlow20 scenario. We show scaled densities of partitions in years 23 and 40 of one simulation replicate. MaleFlow20 uses nucleus and multiplier males in the multiplier. Trait 1 is measured in the nucleus and the multiplier, while trait 2 is measured only in the nucleus. Black vertical lines represent the nucleus mean breeding value for a trait in a year.

## Data Availability

Project name: AlphaPart. Project home page: https://cran.r-project.org/package=AlphaPart. Operating system(s): Windows, MacOS, Linux. Programming language: R & C++. License: GPL-2 | GPL-3. Any restrictions to use by non-academics:-
