# Supplementary material for: AlphaPart—R implementation of the method for partitioning genetic trends
Source: Genet Sel Evol. 2021 Mar 18;53:30. doi: 10.1186/s12711-021-00600-x (PMC7977322; doi:10.1186/s12711-021-00600-x)
Supplement: Supplementary file 1 — Additional file 1: Figure S1. Example session of analysing a breeding programme using import—simulation details. [file 12711_2021_600_MOESM1_ESM.docx]

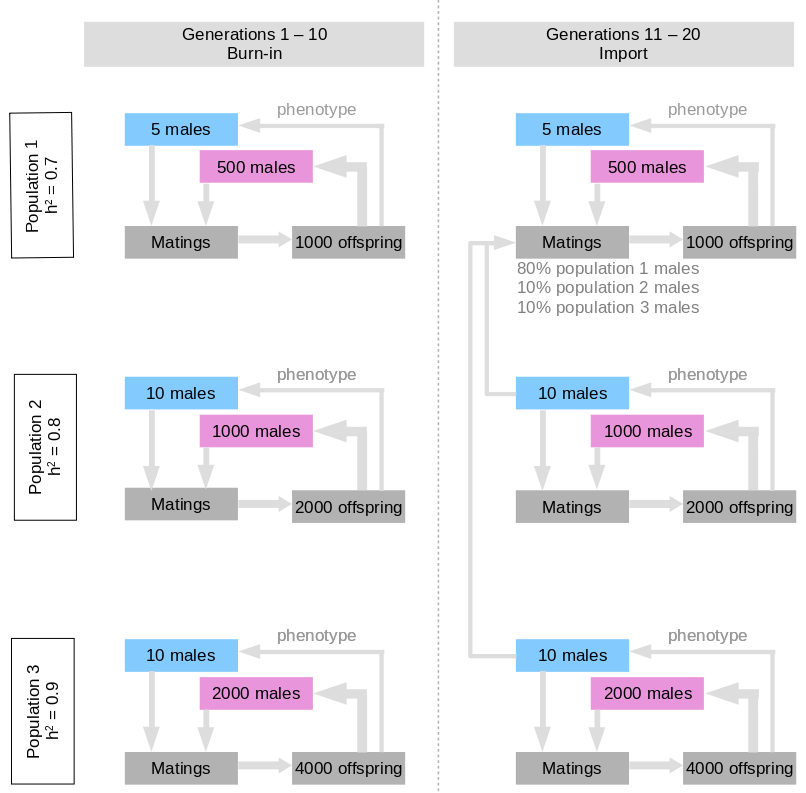


**Figure S1 Example session of analysing a breeding programme using import – simulation details.** We used AlphaSimR (Gaynor et al., 2020) and Markovian Coalescent Simulator (MaCS) to simulation a founder population of 3525 individuals with 10 chromosomes, 1000 segregating sites per chromosome and generic species history. We simulated three additive genetic traits, each controlled with 1000 causal loci, mean 0 and additive genetic variance 1, and heritability of 0.7 for trait 1, 0.8 for trait 2 and 0.9 for trait 3. We set the genetic correlation of traits 1 and 2 to 0.9, of traits 1 and 3 to 0.8, and of traits 2 and 3 to 0.8. We initiated the breeding programme by randomly allocating the founder individuals to three populations: population 1 with 5 males and 500 females, population 2 with 10 males and 1000 females, and population 3 with 10 males and 2000 females. We next ran 10 generation of burn-in where we performed selection only of males and mated the females within each of the populations. Each year we randomly mated all the females with the best allocated number of males and produced two progenies per cross. We then selected males with the best phenotypic values as the fathers of the next generation. We selected males in population 1, population 2, and population 3 based on phenotype values for trait 1, trait 2, and trait 3, respectively. We next ran 10 generations of selection in which we imported males from populations 2 and 3 into population 1. We used the imported males for 20% of the crosses in population 1, 10% from each of the populations 2 and 3. The selection within each of the populations remained unchanged to the burn-in.
