# Supplementary material for: AlphaPart—R implementation of the method for partitioning genetic trends
Source: Genet Sel Evol. 2021 Mar 18;53:30. doi: 10.1186/s12711-021-00600-x (PMC7977322; doi:10.1186/s12711-021-00600-x)
Supplement: Supplementary file 2 — Additional file 2: Figure S2. Distribution of true breeding values and their partitions by trait, year, and tier in the MaleFlow100 scenario. We show scaled densities of partitions in years 23 and 40 of one simulation replicate. MalerFlow100 uses only nucleus males in the multiplier. Trait 1 is measured in the nucleus and the multiplier, while trait 2 is measured only in the nucleus. Black vertical lines represent the nucleus mean breeding value for a trait in a year. [file 12711_2021_600_MOESM2_ESM.docx]

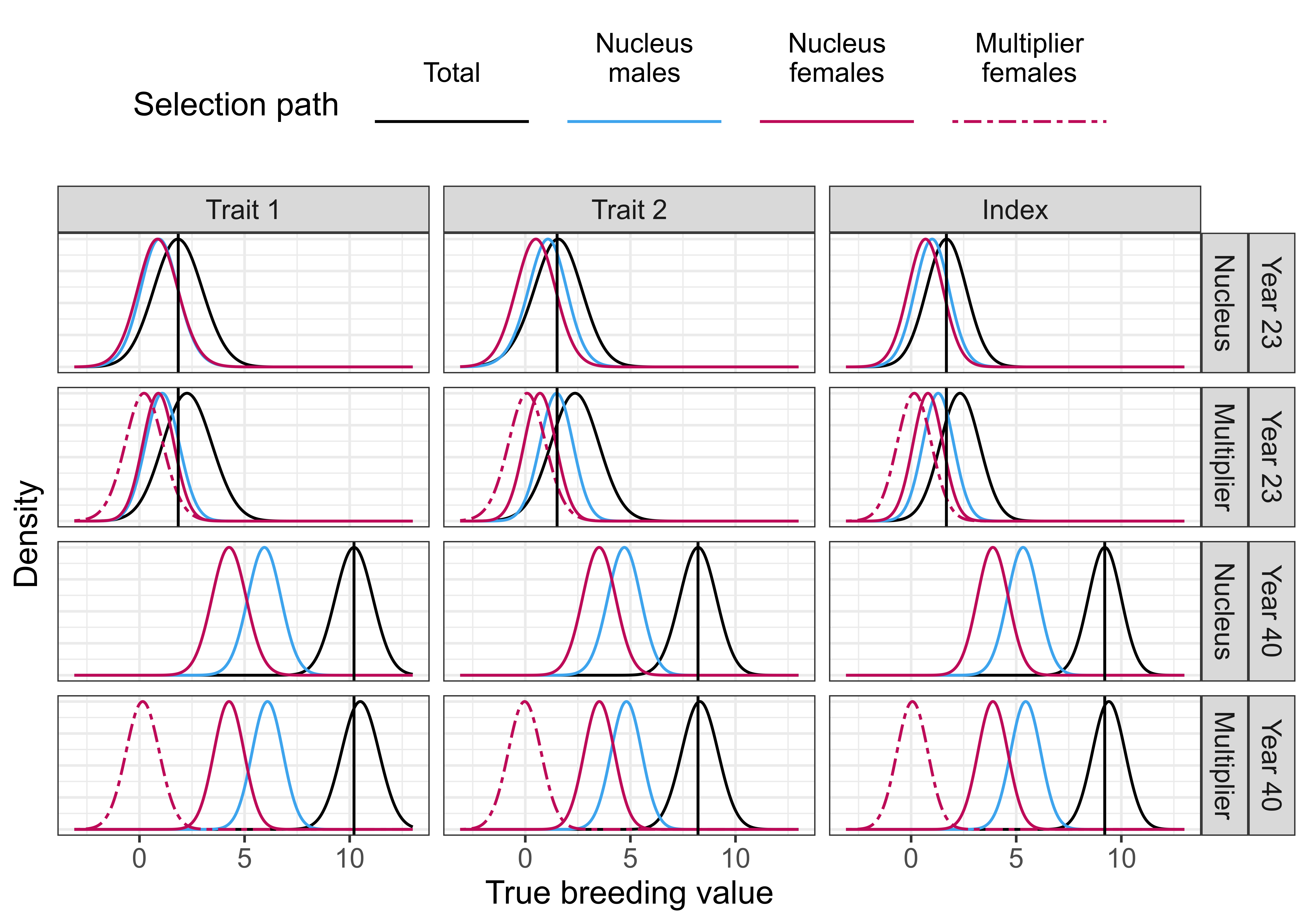


**Figure S2 Distribution of true breeding values and their partitions by trait, year, and tier in the MaleFlow100 scenario.** We show scaled densities of partitions in years 23 and 40 of one simulation replicate. MalerFlow100 uses only nucleus males in the multiplier. Trait 1 is measured in the nucleus and the multiplier, while trait 2 is measured only in the nucleus. Black vertical lines represent the nucleus mean breeding value for a trait in a year.
