# Supplementary material for: AlphaPart—R implementation of the method for partitioning genetic trends
Source: Genet Sel Evol. 2021 Mar 18;53:30. doi: 10.1186/s12711-021-00600-x (PMC7977322; doi:10.1186/s12711-021-00600-x)
Supplement: Supplementary file 3 — Additional file 3: Figure S3. Distribution of true breeding values and their partitions by trait, year, and tier in the MaleFlow20 scenario. We show scaled densities of partitions in years 23 and 40 of one simulation replicate. MaleFlow20 uses nucleus and multiplier males in the multiplier. Trait 1 is measured in the nucleus and the multiplier, while trait 2 is measured only in the nucleus. Black vertical lines represent the nucleus mean breeding value for a trait in a year. [file 12711_2021_600_MOESM3_ESM.docx]

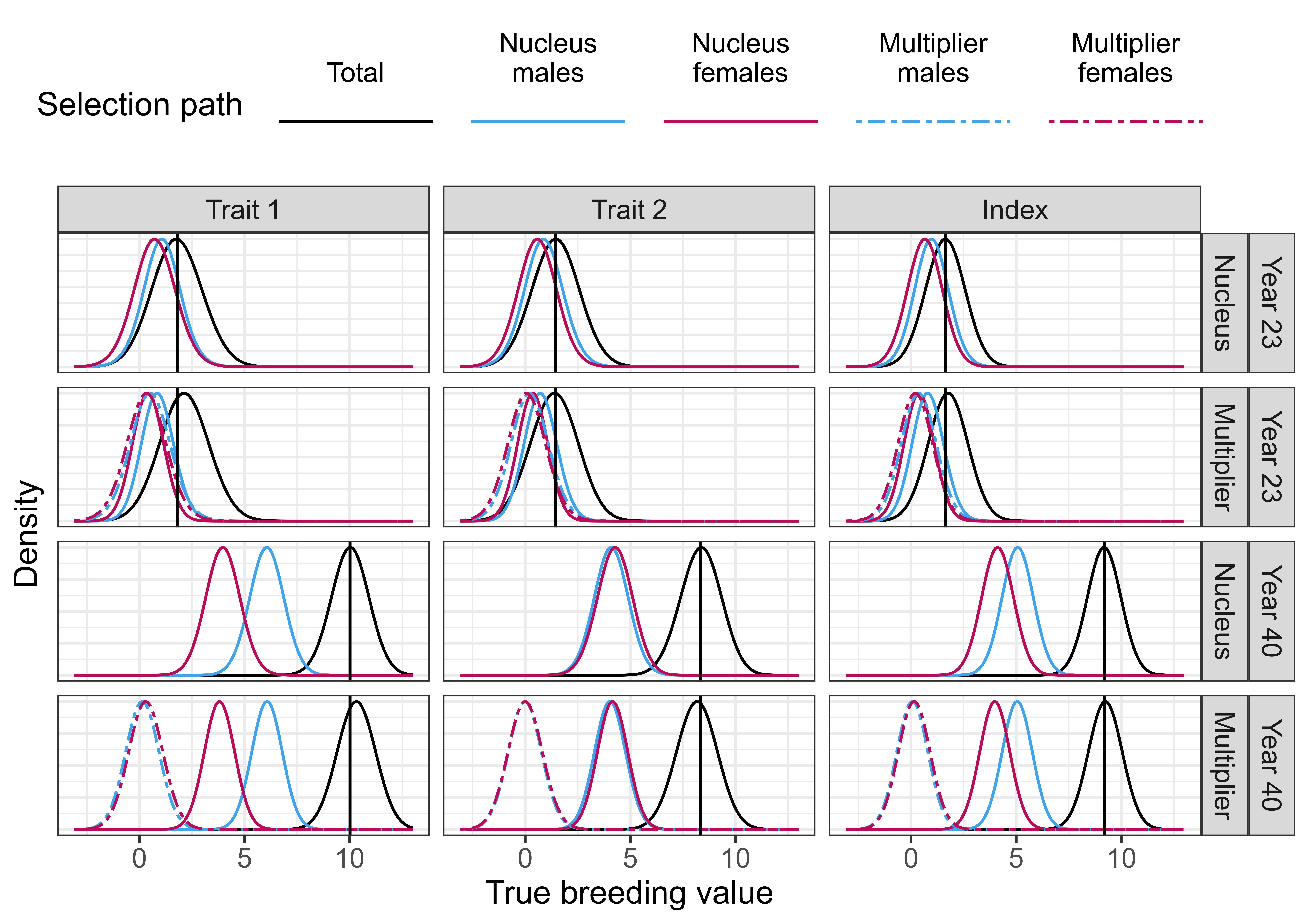


**Figure S3 Distribution of true breeding values and their partitions by trait, year, and tier in the MaleFlow20 scenario.** We show scaled densities of partitions in years 23 and 40 of one simulation replicate. MalerFlow20 uses nucleus and multiplier males in the multiplier. Trait 1 is measured in the nucleus and the multiplier, while trait 2 is measured only in the nucleus. Black vertical lines represent the nucleus mean breeding value for a trait in a year.
